# Nationwide trends in prevalence of underweight, overweight, and obesity among people with disabilities in South Korea from 2008 to 2017

**DOI:** 10.1038/s41366-021-01030-x

**Published:** 2021-12-03

**Authors:** Dong-Hwa Lee, So Young Kim, Jong Eun Park, Hyun Jeong Jeon, Jong-Hyock Park, Ichiro Kawachi

**Affiliations:** 1 Department of Internal Medicine, Chungbuk National University College of Medicine and Chungbuk National University Hospital, Cheongju, Korea; 2 Department of Public Health and Preventive Medicine, Chungbuk National University Hospital, Cheongju, Korea; 3 College of Medicine, Chungbuk National University, Cheongju, Korea; 4 Institute of Health & Science Convergence, Chungbuk National University, Cheongju, Korea; 5 Harvard T.H. Chan School of Public Health, Harvard University, Boston, MA USA

**Keywords:** Risk factors, Health policy, Epidemiology

## Abstract

**Objectives:**

This study investigated the 10-year trends of weight and prevalence of underweight, overweight and obesity according to disability grade and types compared with those without disabilities.

**Methods:**

This serial cross-sectional analysis was conducted using national disability registration data with national general health checkup data from 2008 to 2017. Age-standardized prevalence of underweight and obesity were analyzed for each year, according to the presence, type, and severity of disabilities. Odds of underweight, overweight, obesity, and severe obesity were examined by multinomial logistic regression after adjusting for socio-demographic and clinical variables using data in 2017.

**Results:**

Over 10 million subjects in each year were included in the analysis. In 2017, 14,246,785 people with age between 19 and 110 years were included and 53.1% was men. For 10 years, age-standardized prevalence of obesity and severe obesity showed significant increases regardless of sex and presence of disability. However, age-standardized underweight prevalence in people without disability tended to decrease whereas it was an increase in 2012 and the prevalence has remained steady since in people with disability. People with disabilities had higher odds of underweight compared to those without disability (OR 1.41, 95% CI 1.38–1.44 in male and OR 1.31, 95% CI 1.28–1.34 in female), especially in those with severe disabilities (OR 2.00, 95% CI 1.94–2.06 in male and OR 1.83, 95% CI 1.77–1.89 in female). Women with disabilities are more likely to be obese than those without disabilities regardless of disability severity (OR 1.40, 95% CI 1.38–1.41). Participants with mental disorder showed the highest prevalence of obesity, followed by epilepsy and developmental disability.

**Conclusions:**

Having a disability was associated with higher odds/probability of both obesity and underweight. The intersection of female, severe disability, and mental/developmental disabilities was associated with probability of severe obesity. Simultaneous efforts are needed to develop health policy to reduce both the prevalence of obesity and underweight.

## Introduction

Maintenance of optimal body weight is crucial for disease prevention and preserving quality of life. Obesity is strongly associated with chronic diseases including metabolic syndrome, type 2 diabetes mellitus, hypertension, hyperlipidemia, and cardiovascular disease [1]. As a result, individuals with obesity suffer a higher burden of morbidity and mortality rate compared to individuals who are not obese [2]. At the same time, underweight is also related to elevated all-cause mortality and diseases such as osteoporosis, sarcopenia, low fertility, and anemia [3–6].

The prevalence of obesity has increased rapidly in recent decades worldwide to the point that it now represents the fourth leading cause of the global burden of disease [7, 8]. Increasing prevalence of obesity is also observed in Korea especially for men. According to the data from the 2015 Korea National Health and Nutrition Examination Survey, the age-standardized prevalence of obesity increased from about 25% in 1998 to 40% in 2015 for men whereas it was still similar about 26% for women [9].

At the same time, one in three people in the world suffer from food insecurity and under-weight [10]. Underweight or undernutrition are often ignored or overshadowed in high-income countries because its prevalence is very low. However, the prevalence of underweight or malnutrition still remains high, especially in vulnerable population such as children, adolescents, pregnant women, older adults, and people with disabilities, even in high-income countries [9, 11].

Previous studies have primarily focused on people with specific type of disabilities such as intellectual disability, physical disability, and spinal cord injury, or used self-reported information on weight, disability type and severity [12–16]. There are few studies evaluating the weight distribution in whole disability population as well as long term trends.

In Korea, all people are covered by universal health insurance regardless of income level or health risk. In addition, the national disability registration system defines the type and severity of disabilities based on medical examination and specific criteria, so that the information is linked to welfare benefits. This system provides a unique opportunity to evaluate the prevalence of obesity according to the types of disabilities and their severity. Using these linked data, we investigated the trends in weight and prevalence of underweight, overweight and obesity according to disability severity and type over a 10-year observation period (2007-2017).

## Methods

### Data sources and study population

We linked national disability registration data with the general health checkup database of the National Health Information Database (NHID) in South Korea. A flowchart of the study population selection process is presented in Supplementary Fig. 1. The NHID is a public database on health care utilization, health screening, socio-demographic variables, and mortality for the whole population of South Korea, maintained by the Korean National Health Insurance Service (NHIS) [17]. From these data, we extracted information on health screening, comorbidities, and socio-demographic variables including age, sex, type of insurance, insurance premium, and residential area. The general health checkup for adults older than 19 years forms a part of the national health screening strategy, which is provided free every other year to all citizens [18]. The participation rate in general health checkup was 65.3% in 2008 and increased to 78.5% in 2017. From these examination, we extracted individual information on anthropometry and health behaviors including smoking status, alcohol consumption, physical activity, and walking.

Using the national disability registration data, we collected information on disability by type and severity level. The database covers 93.8% of the total population with disabilities in 2011 [19]. Using the Korean personal identification number, the disability types and severity levels were linked with the variables selected from the NHID as mentioned above. Data from a total of 123,334,034 subjects who participated in the general health checkup program between 2008 and 2017 were analyzed.

### Definition of underweight, obesity, and other variables

We extracted body weight, height, and waist circumference from the results of the general health checkup. Body mass index (BMI) was calculated as the ratio of weight in kilograms to height in meters squared (kg/m^2^). On the basis of the WHO Asia-Pacific regional guidelines, we defined the cut-offs for underweight (<18.5 kg/m^2^), overweight (≥23.0 kg/m^2^), and obesity (≥25.0 kg/m^2^), and severe obesity (≥30.0 kg/m^2^), respectively [20, 21]. Abdominal obesity was defined as waist circumference ≥90 cm for men and ≥85 cm for women using the cut-off in Korea [22].

The national disability registration data defines fifteen categories of disability [23]. Disability severity is officially graded from 1 (very severe) to 6 (very mild) based on functional losses and clinical impairment as determined by a medical specialist. In the present study, disability severity was classified as severe (grade 1-3) or mild (grade 4-6).

Other variables collected from the NHIS included age, sex, type of insurance, insurance premium, residential area, and comorbidities. As a proxy measure for actual household income, we used the insurance premium categories “quartile” and “Medical Aid beneficiaries”, as provided by the NHIS: Medical Aid (lowest), first quartile, second quartile, third quartile, and fourth quartile (highest). Insurance premiums are calculated based on income, property, and automobile taxes for each household [24]. Residential areas were grouped into three categories, metropolitan, urban, and rural, based on Korean ZIP code. The Charlson comorbidity index was used to group study subjects into four categories based on the index score: 0, 1–2, 3–4, and ≥5 (the most severe) [25]. In the drinking habits, heavy use of alcohol was defined as 7 or more alcoholic drinks for males or 5 or more alcoholic drinks for females on a single occasion at least twice a week. Physical activity was derived based on questions about how many days per week participants participated in physical activity (moderate-intensity activity or walking) for more than 30 minutes.

### Statistical analyses

The general characteristics of the subjects were analyzed using descriptive statistics. Mean BMI and waist circumference according to sex and disability were calculated using Student’s t-test or one-way analysis of variance. Trends in age-standardized prevalence of underweight, and obesity, severe obesity, and abdominal obesity were calculated, using the direct standardization method. An age-standardized rate is a weighted average of crude age-specific rates, where the crude rates are calculated for different age groups and the weights are the proportions of persons in the corresponding age groups of a standard population [26]. In this study, age-standardization was performed using the age structure of general population in the 2005 Population and Housing Census of Korea as the standard population. To examine the association between disability and underweight/overweight/obesity and severe obesity, we developed a multinomial logistic regression adjusting for age, income level, residence, health behaviors (smoking, alcohol, moderate physical activity, walking), and Charlson comorbidity index, using the most recent dataset available (2017). All analyses were performed using SAS software ver. 9.4 (SAS Institute Inc., Cary, NC, USA). Two-sided p-values of 0.05 were considered significant.

### Ethical considerations

Data were anonymized by the data holders before being accessed by the research team. Ethics approval was granted by the International Review Board of Chungbuk National University (IRB No. CBNU-202010-HRHR-0171).

## Results

### Study participants

During the period 2008–2017, over 10 million subjects in each year performed the general health checkup of the NHIS. The number of subjects by disability, sex, and age group are shown in Table 1. In terms of age distribution, about half of the subjects were in age group between 40 and 59 years.

**Table 1:** Baseline characteristics of the study population.

	Year									
	2008	2009	2010	2011	2012	2013	2014	2015	2016	2017
All subjects	10,207,865	10,496,170	11,453,541	11,718,395	12,188,101	12,319,408	13,244,363	13,469,135	13,990,271	14,246,785
Disability status										
Without disability	9,710,285	9,926,375	10,830,543	11,086,412	11,504,153	11,636,495	12,530,563	12,765,968	13,261,625	13,517,497
	(95.1)	(94.6)	(94.6)	(94.6)	(94.4)	(94.5)	(94.6)	(94.8)	(94.8)	(94.9)
With disability	497,580	569,795	622,998	631,983	683,948	682,913	713,800	703,167	728,646	729,288
	(4.9)	(5.4)	(5.4)	(5.4)	(5.6)	(5.5)	(5.4)	(5.2)	(5.2)	(5.1)
Sex										
Male	5,738,862	5,791,481	6,300,465	6,429,697	6,643,968	6,709,117	7,177,549	7,239,196	7,464,340	7,566,776
	(56.2)	(55.2)	(55.0)	(54.9)	(54.5)	(54.5)	(54.2)	(53.8)	(53.4)	(53.1)
Female	4,469,003	4,704,689	5,153,076	5,288,698	5,544,133	5,610,291	6,066,814	6,229,939	6,525,931	6,680,009
	(43.8)	(44.8)	(45.0)	(45.1)	(45.5)	(45.5)	(45.8)	(46.3)	(46.7)	(46.9)
Age, years										
Range	19–110	19–116	19–122	19–118	19–114	19–114	19–120	19–116	19–116	19–110
19–29	1,424,378	1,313,736	1,241,901	1,259,490	1,167,965	1,214,250	1,230,466	1,198,815	1,281,173	1,271,028
	(14.0)	(12.5)	(10.8)	(10.8)	(9.6)	(9.9)	(9.3)	(8.9)	(9.2)	(8.9)
30–39	2,156,463	2,007,275	2,233,844	2,209,935	2,255,245	2,232,117	2,374,293	2,345,681	2,317,242	2,317,560
	(21.1)	(19.1)	(19.5)	(18.9)	(18.5)	(18.1)	(17.9)	(17.4)	(16.6)	(16.3)
40–49	2,674,400	2,737,581	3,012,854	3,073,045	3,148,862	3,231,269	3,401,871	3,458,637	3,533,803	3,546,072
	(26.2)	(26.1)	(26.3)	(26.2)	(25.8)	(26.2)	(25.7)	(25.7)	(25.3)	(24.9)
50–59	2,048,933	2,220,661	2,584,978	2,700,286	2,937,737	2,901,130	3,188,895	3,266,819	3,374,367	3,443,253
	(20.1)	(21.2)	(22.6)	(23.0)	(24.1)	(23.6)	(24.1)	(24.3)	(24.1)	(24.2)
60–69	1,252,670	1,421,821	1,514,732	1,520,986	1,638,667	1,660,530	1,865,996	1,991,055	2,173,122	2,276,851
	(12.3)	(13.6)	(13.2)	(13.0)	(13.4)	(13.5)	(14.1)	(14.8)	(15.5)	(16.0)
70–79	566,654	687,709	741,936	812,388	883,009	904,229	974,985	975,049	1,050,569	1,101,433
	(5.6)	(6.6)	(6.5)	(6.9)	(7.2)	(7.3)	(7.4)	(7.2)	(7.5)	(7.7)
≥80	84,367	107,387	123,296	142,265	156,616	175,883	207,857	233,079	259,995	290,588
	(0.8)	(1.0)	(1.1)	(1.2)	(1.3)	(1.4)	(1.6)	(1.7)	(1.9)	(2.0)

Values are expressed as number (%).

### Characteristics of people with and without disabilities

The number of individuals without and with disabilities was 13,517,497 and 729,288, respectively. A summary of the general characteristics of the two groups is shown in Table 2 as of 2017. Among people with disabilities, 27.5% had severe disabilities with the most frequent type being physical disability (60.3%). Male were significantly more represented among people with disabilities (61.8% vs. 52.7%, *P* < 0.001). People with disabilities were also older (60.7 ± 13.5 years in with disability group and 49.0 ± 14.1 years in without disability group, *P* < 0.001). The disability group was more likely to report lower incomes and live in a rural area and had more comorbidities as assessed by Charlson comorbidity index. There were also significant differences between two groups in lifestyle parameters including smoking, alcohol, and physical activity.

**Table 2:** Characteristics of the population with and without disabilities in the most recent year with available data (2017).

	Without disability (*n* = 13,517,497)		With disability (*n* = 729,288)		*P-* value
Disability severity					
Severe (grades 1–3)			200,249	(27.5)	
Mild (grades 4–6)			529,039	(72.5)	
Disability type					
Physical			440,057	(60.3)	
Brain injury			41,288	(5.7)	
Facial			963	(0.1)	
Visual			78,005	(10.7)	
Hearing			84,212	(11.6)	
Language			4694	(0.6)	
Intellectual			33,234	(14.6)	
Autism			965	(0.1)	
Mental			18,023	(2.5)	
Renal disease			14,962	(2.1)	
Heart disease			1646	(0.2)	
Respiratory disease			2811	(0.4)	
Liver disease			3070	(0.4)	
Ostomy			3544	(0.5)	
Epilepsy			1814	(0.3)	
Sex					<0.001
Male	7,116,431	(52.7)	450,345	(61.8)	
Female	6,401,066	(47.4)	278,943	(38.3)	
Age, years					
All subjects	49.0 ± 14.1		60.7 ± 13.5		<0.001
19–29	1,256,863	(9.3)	14,165	(1.9)	<0.001
30–39	2,284,457	(16.9)	33,103	(4.5)	
40–49	3,450,796	(25.5)	95,276	(13.1)	
50–59	3,268,245	(24.2)	175,008	(24.0)	
60–69	2,077,870	(15.4)	198,981	(27.3)	
70–79	944,474	(7.0)	156,959	(21.5)	
≥80	234,792	(1.7)	55,796	(7.7)	
Type of insurance					<0.001
Health insurance insured	13,428,680	(99.3)	670,154	(91.9)	
Medical aid	88,817	(0.7)	59,134	(8.1)	
Income level					<0.001
Medical aid (lowest)	88,817	(0.7)	59,134	(8.1)	
First quartile	2,309,409	(17.1)	157,459	(21.6)	
Second quartile	2,994,256	(22.2)	133,103	(18.3)	
Third quartile	3,631,763	(26.9)	160,809	(22.1)	
Fourth quartile (highest)	4,149,992	(30.7)	208,463	(28.6)	
Unknown	343,260	(2.5)	10,320	(1.4)	
Residence					<0.001
Metropolitan	8,384,232	(62.0)	392,753	(53.9)	
City	3,961,235	(29.3)	231,531	(31.8)	
Rural	1,169,144	(8.7)	105,004	(14.4)	
Unknown	2,886	(0.02)	0	(0.0)	
Smoking					<0.001
Non-smoker	8,234,268	(60.9)	433,256	(59.4)	
Ex-smoker					
<20 pack-years	1,678,314	(12.4)	86,644	(11.9)	
≥20 pack-years	665,448	(4.9)	69,649	(9.6)	
Unknown pack-years	5606	(0.04)	328	(0.04)	
Current smoker					
<20 pack-years	1,996,594	(14.8)	70,605	(9.7)	
≥20 pack-years	930,990	(6.9)	68,527	(9.4)	
Unknown pack-years	4009	(0.03)	179	(0.02)	
Unknown smoking status	2268	(0.02)	100	(0.01)	
Alcohol					<0.001
None	6,681,609	(49.4)	473,412	(64.9)	
Social	4,801,342	(35.5)	173,213	(23.8)	
Heavy	2,022,260	(15.0)	82,148	(11.3)	
Unknown	12,286	(0.1)	515	(0.07)	
Moderate physical activity of ≥30 minutes per day					<0.001
None	6,359,219	(47.0)	432,780	(59.3)	
<5 days/week	5,905,506	(43.7)	224,030	(30.7)	
≥ 5days/week	1,248,291	(9.2)	72,220	(9.9)	
Unknown	4,481	(0.03)	258	(0.04)	
Walking of ≥30 minutes per day					<0.001
None	2,858,495	(21.2)	227,409	(31.2)	
<5 days/week	5,977,290	(44.2)	266,747	(36.6)	
≥ 5days/week	4,676,223	(34.6)	234,810	(32.2)	
Unknown	5,489	(0.04)	322	(0.04)	
Charlson comorbidity index					<0.001
0	7,071,628	(52.3)	217,423	(29.8)	
1–2	5,003,079	(37.0)	299,823	(41.1)	
3–4	1,100,577	(8.1)	139,349	(19.1)	
≥5	342,213	(2.5)	72,693	(10.0)	

Values are expressed as mean ± SD and number (%).

*P*-values were calculated using Student’s *t*-test for continuous data and the chi-squared test for categorical data.

### Changes in BMI and waist circumference from 2008 to 2017

**Fig. 1:**
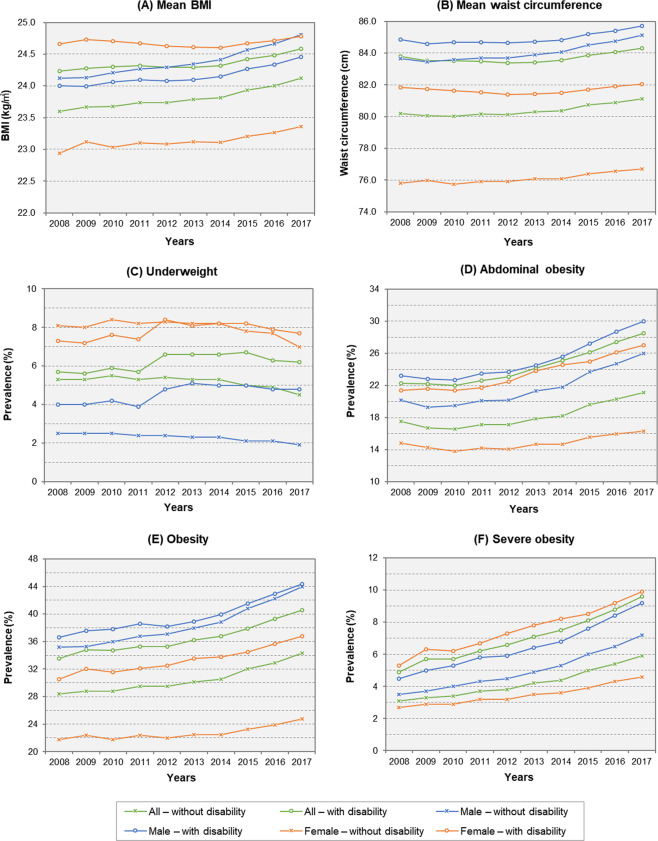
Trends in anthropometric measures and age-standardized prevalence of underweight, obesity, and severe obesity according to sex and disability during 2008–2017. Mean (**A**) BMI and (**B**) waist circumference by sex and disability characteristics. Age-standardized prevalence of **C** underweight, **D** abdominal obesity, **E** obesity, and **F** severe obesity by sex and disability characteristics. BMI body mass index.

Secular changes in mean BMI and waist circumference of the subjects according to sex and with or without disabilities are presented in Fig. 1A, B and Supplementary Table 1. Overall, both mean BMI and waist circumference increased significantly during the 10-year period in all groups (*P* for trend <0.001 in all groups). People with disabilities had higher BMI and waist circumference than those without disabilities throughout all observed years. The mean BMI in people with disabilities was 24.2 ± 3.3 kg/m^2^ in 2008 and 24.6 ± 3.6 kg/m^2^ in 2017. Among people without disabilities, the mean BMI also increased from 23.6 ± 3.2 kg/m^2^ in 2008 to 24.1 ± 3.5 kg/m^2^ in 2017. Different patterns were observed in BMI especially when subgroup analysis was performed by sex. Female with disabilities showed higher BMI compared to those without disabilities. However, among male, the mean BMI was lower for those with disabilities compared to male without disabilities. We also analyzed mean BMI and waist circumference in each year according to severity and type of disability and the results are summarized in Supplementary Table 1.

### Changes in prevalence according to weight category for 10 years

Trends in age-standardized prevalence of underweight, obesity, severe obesity and abdominal obesity from 2008 to 2017 are shown in Fig. 1C–F and Supplementary Table 2. Age-standardized underweight prevalence in people without disability tended to decrease from 5.3% in 2008 to 4.5% in 2017. By contrast, among people with disabilities, there was an increase in 2012 and the prevalence has remained steady since. The underweight prevalence was higher in female than male regardless of disability.

Age-standardized prevalence of obesity, severe obesity, and abdominal obesity showed significant increases in all groups over the 10 years. In particular, the obesity prevalence in female with and without disabilities showed a wide gap, and the gap gradually widened from 8.7% in 2008 to 12.0% in 2017. Female with disabilities showed the highest prevalence of severe obesity during the observation period, whereas female without disabilities exhibited the lowest prevalence of obesity. The disparity in severe obesity widened significantly over time from 2.6% in 2008 to 5.3% in 2017. By contrast, obesity rates in male with and without disabilities have converged over time. However, the gap in severe obesity prevalence between male with and without disabilities gradually increased as in female.

### Prevalence of underweight and obesity according to severity and type of disability

**Fig. 2:**
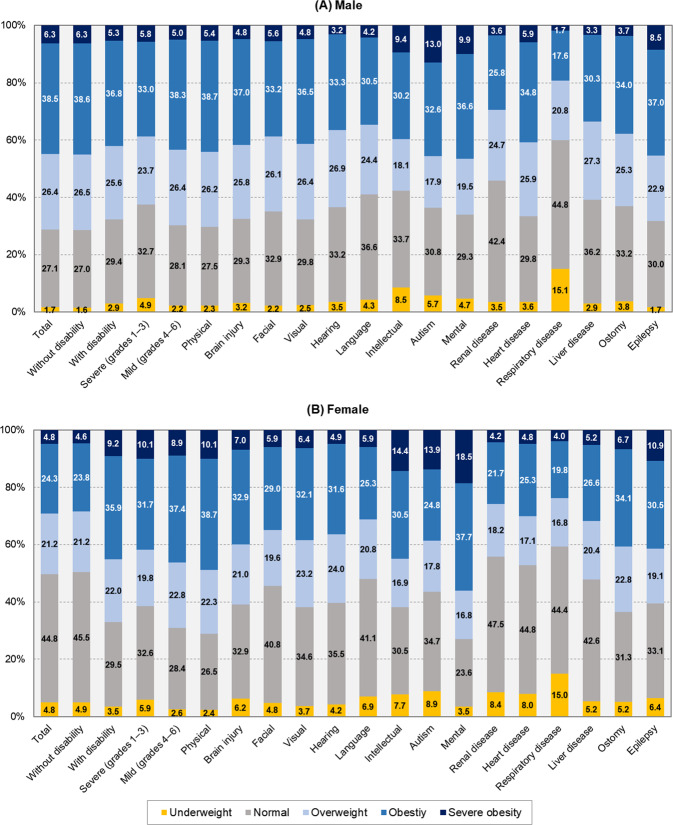
BMI distribution according to disability, disability severity and type by sex in the most recent year with available data (2017). Percentage of BMI categories by disability characteristics in **A** men and **B** women. BMI body mass index.

Distribution of five BMI categories (underweight, normal, overweight, obesity, and severe obesity) according to disability severity and type by sex the most recent year with available data (2017) are presented in Fig. 2. Age-standardized prevalence of underweight, obesity, severe obesity, and abdominal obesity by disability grade and type are presented in Supplementary Table 3. The underweight prevalence was significantly higher in male with severe disabilities compared to those with mild disabilities (6.9% vs. 2.6%). By contrast, female with disabilities had a high underweight rate regardless of disability severity (8.5% for female with severe disabilities vs. 7.5% for female with mild disabilities, respectively).

Female with severe disabilities (grades 1–3) also showed higher prevalence of obesity than those with mild disabilities (39.2% vs. 31.8%). By contrast, obesity was higher in participants with mild disabilities than those with severe disabilities in male (46.9% vs. 40.3%, respectively). For most types of disabilities, male had a higher obesity prevalence than female, whereas in people with mental and developmental disabilities, the pattern was reversed. Regarding prevalence of abdominal obesity, similar trends were observed as the above. Crude rates of underweight and obesity were shown in Supplementary Table 4.

### Odds of underweight and obesity by disability

The multinomial logistic regression analysis results are displayed by disability grade and type in Table 3. In terms of the underweight prevalence, people with disability had higher odds (both in males and females) compared to those without disability (OR 1.41, 95% CI 1.38–1.44, and OR 1.31, 95% CI 1.28–1.34, respectively). Individuals with disability showed significantly higher odds for underweight regardless of the disability severity. All types of disability, except facial disability, were associated with increased probability of underweight.

**Table 3:** Multinomial logistic regression for the categories of BMI in the most recent year with available data (2017).

	Underweight^a^ (BMI < 18.5 kg/m^2^)	Overweight^a^ (23.0≤ BMI < 25.0 kg/m^2^)	Obesity^a^ (25.0≤ BMI < 30.0 kg/m^2^)	Severe obesity^a^ (BMI ≥ 30.0 kg/m^2^)
	OR (95% CI)^b^	OR (95% CI)^b^	OR (95% CI)^b^	OR (95% CI)^b^
***Male***				
Disability				
Without disability	1.00	1.00	1.00	1.00
With disability	1.41 (1.38–1.44)	0.93 (0.92–0.94)	0.95 (0.95–0.96)	1.05 (1.04–1.07)
Disability severity				
Without disability	1.00	1.00	1.00	1.00
Severe (grades 1–3)	2.00 (1.94–2.06)	0.80 (0.79–0.82)	0.79 (0.78–0.80)	0.92 (0.90–0.95)
Mild (grades 4–6)	1.12 (1.09–1.15)	0.99 (0.98–0.99)	1.03 (1.02–1.04)	1.12 (1.10–1.14)
Disability type				
Without disability	1.00	1.00	1.00	1.00
Physical	1.23 (1.20–1.27)	0.99 (0.98–1.00)	1.03 (1.02–1.04)	1.16 (1.14–1.18)
Brain injury	1.43 (1.33–1.54)	0.94 (0.90–0.97)	0.95 (0.92–0.98)	0.94 (0.88–1.00)
Facial	1.08 (0.62–1.90)	0.82 (0.66–1.01)	0.72 (0.59–0.88)	0.81 (0.56–1.18)
Visual	1.18 (1.11–1.25)	0.95 (0.92–0.97)	0.95 (0.93–0.97)	0.99 (0.95–1.04)
Hearing	1.22 (1.16-1.28)	0.93 (0.91–0.96)	0.91 (0.89–0.93)	0.85 (0.81–0.89)
Language	1.73 (1.46–2.05)	0.70 (0.65–0.77)	0.61 (0.56–0.66)	0.57 (0.48–0.68)
Intellectual	3.27 (3.01–3.46)	0.65 (0.63–0.68)	0.75 (0.72–0.77)	1.02 (0.97–1.08)
Autism	2.18 (1.61–2.97)	0.84 (0.69–1.02)	1.17 (0.99–1.38)	1.46 (1.17–1.83)
Mental	1.95 (1.76–2.16)	0.78 (0.74–0.83)	1.01 (0.96–1.06)	1.63 (1.51–1.77)
Renal disease	1.24 (1.11–1.39)	0.56 (0.53–0.59)	0.37 (0.35–0.39)	0.29 (0.26–0.33)
Heart disease	1.61 (1.16–2.24)	0.91 (0.78–1.06)	0.88 (0.75–1.02)	1.17 (0.89–1.53)
Respiratory disease	4.53 (3.98–5.15)	0.48 (0.43–0.54)	0.29 (0.26–0.33)	0.26 (0.18–0.36)
Liver disease	1.40 (1.09–1.80)	0.70 (0.63–0.77)	0.50 (0.45–0.56)	0.38 (0.30–0.47)
Ostomy	1.38 (1.10–1.73)	0.83 (0.75–0.93)	0.83 (0.75–0.92)	0.83 (0.66–1.05)
Epilepsy	0.79 (0.48–1.31)	0.83 (0.70–0.99)	0.91 (0.78–1.06)	1.25 (0.98–1.60)
***Female***				
Disability				
Without disability	1.00	1.00	1.00	1.00
With disability	1.31 (1.28–1.34)	1.12 (1.10–1.12)	1.40 (1.38–1.41)	2.08 (2.05–2.11)
Disability severity				
Without disability	1.00	1.00	1.00	1.00
Severe (grades 1–3)	1.83 (1.77–1.89)	1.02 (1.00–1.05)	1.29 (1.27–1.32)	2.10 (2.05–2.16)
Mild (grades 4–6)	1.05 (1.02–1.08)	1.14 (1.13–1.16)	1.43 (1.42–1.45)	2.07 (2.04–2.11)
Disability type				
Without disability	1.00	1.00	1.00	1.00
Physical	1.07 (1.04–1.11)	1.20 (1.18–1.22)	1.59 (1.57–1.61)	2.51 (2.46–2.56)
Brain injury	2.10 (1.95–2.25)	0.88 (0.84–0.92)	0.99 (0.95-1.03)	1.13 (1.05-1.21)
Facial	1.37 (0.84-2.25)	0.85 (0.64–1.13)	1.05 (0.81–1.35)	1.15 (0.74–1.81)
Visual	1.23 (1.16–1.32)	1.02 (0.99–1.05)	1.10 (1.06–1.13)	1.29 (1.23–1.36)
Hearing	1.21 (1.14–1.28)	1.00 (0.97–1.03)	1.01 (0.98–1.04)	0.96 (0.91–1.01)
Language	2.00 (1.59–2.53)	0.83 (0.71-0.97)	0.80 (0.69-0.92)	0.96 (0.75-1.23)
Intellectual	1.82 (1.70–1.96)	1.37 (1.30–1.44)	2.17 (2.07–2.27)	4.04 (3.81–4.27)
Autism	1.20 (0.58–2.50)	2.22 (1.25–3.94)	3.17 (1.89–5.35)	5.21 (2.79–9.72)
Mental	1.75 (1.55–1.97)	1.28 (1.20–1.37)	2.43 (2.30–2.57)	5.71 (5.35–6.10)
Renal disease	2.24 (2.03–2.47)	0.57 (0.53–0.61)	0.48 (0.45–0.52)	0.41 (0.36–0.47)
Heart disease	2.01 (1.45–2.79)	0.55 (0.43–0.70)	0.59 (0.48–0.73)	0.60 (0.40–0.90)
Respiratory disease	4.90 (3.92–6.13)	0.52 (0.42–0.64)	0.45 (0.37–0.55)	0.49 (0.33–0.72)
Liver disease	1.71 (1.23–2.38)	0.69 (0.57–0.84)	0.68 (0.57–0.81)	0.67 (0.48–0.93)
Ostomy	1.90 (1.47–2.46)	0.96 (0.83–1.12)	1.03 (0.90–1.18)	1.15 (0.91–1.45)
Epilepsy	2.30 (1.71–3.08)	1.05 (0.86–1.27)	1.42 (1.20–1.69)	2.45 (1.93–3.11)

*OR* odds ratio; *CI* confidence interval.

^a^Normal weight (18.5 ≤ BMI < 23.0 kg/m^2^) is the reference group for the model.

^b^Adjust for age, income level, residence, smoking, alcohol, moderate physical activity, walking, and Charlson comorbidity index.

Obesity likelihood showed different patterns according to sex, severity or types of disabilities. In male, after adjusting for socio-demographic and clinical variables, mild disability was associated with slightly increased odds of obesity and severe obesity (OR 1.03, 95% CI 1.02–1.04 and OR 1.12, 95% CI 1.10–1.14, respectively). However, severe disability showed an association with lower prevalence of obesity (OR 0.79, 95% CI 0.78–0.80). On the other hand, female with disabilities are more likely to be obese than those without disabilities regardless of disability severity (OR 1.40, 95% CI 1.38–1.41). The likelihood of severe obesity was more prominent in female with disabilities (OR 2.08, 95% CI 2.05–2.11). Regarding types of disabilities, mental or developmental disabilities including intellectual disorder and autism showed high odds ratio among female with severe obesity (OR 5.71, 95% CI 5.35–6.10 for mental, OR 5.21, 95% CI 2.79–9.72 for autism, and OR 4.04, 95% CI 3.81–4.27 for intellectual, respectively).

## Discussion

We analyzed 10-year trends of weight distribution and found that obesity and underweight were both more prevalent in people with disabilities. The weight of people with disabilities is more skewed to both extremes than that of the non-disabled. Especially, this pattern was more prominent in women with severe disabilities and those with mental, developmental, and physical disabilities.

Underweight was revealed as a significant problem in people with disability in this study. While underweight has tended to decrease over time in individuals without disabilities, the prevalence of underweight was not declined even worsened during the past decade in those with disabilities. The underweight prevalence of the people with disabilities suddenly rose in 2012. This date coincides with the implementation of the national personal assistance service for people with disabilities in October 2011, when mobility for the disabled was improved. The service raised the national health screening rate for people with severe disabilities who have mobility difficulties [27], which we hypothesize to account for the sharp increase in the underweight prevalence (i.e., more detection). The results also imply that screening for more people with severe disabilities could lead to higher underweight rates than now.

The higher prevalence of underweight was especially notable in people with severe disabilities and internal organ impairment affecting the respiratory organs, kidney, liver, and heart. In people with disability, malnutrition and sarcopenia resulting from physical inactivity might contribute to increase probability of underweight. In fact, our results showed that underweight was more frequent in people with severe disability. A previous study found that underweight was more common in both male and female adults with intellectual disability than in the general population [28]. In that study, after controlling for age, it was more problematic in males than in females, in line with our results. However, given the higher underweight rate of women with disabilities for most types of disabilities (except mental disorder), this result requires careful interpretation. Namely, this could be the result of more vulnerable women with mental disorder not being able to participate in health checkups. There has been no previous study on underweight and disability performed across the various types of disability. Several previous studies demonstrated that underweight was associated with morbidity of some disease and economic burden [29–31]. Therefore, future research is needed to focus on underweight in people with disability and policies to reduce the proportion of underweight especially in people with severe disability.

It is widely recognized that obesity is a major public health problem worldwide. However, few studies have evaluated the prevalence of obesity in people with disability [16, 32]. In the present study, mean BMI and waist circumference and the prevalence of obesity, severe obesity, and abdominal obesity increased significantly regardless of sex and presence of disability in South Korea from 2008 to 2017. It is also worth noting the prevalence of obesity was the highest in women with disability during observation periods while women without disability showed the lowest mean BMI and obesity prevalence. As a result, the disparities in mean BMI and obesity prevalence between people with and without disability were more prominent in women than in men. In general population, obesity was more frequently observed in women than men [18, 33]. However, in Korea, the prevalence of obesity was higher in male than in women, and a recent study demonstrated that a decreasing tendency of obesity was observed in Korean women [17]. Nevertheless, higher obesity prevalence is still major problem in women with disabilities. In this study, the proportion of population according to disability type differed by sex. Women had higher prevalence of some types of disability including mental impairment, physical disability, and intellectual disability and autism. In addition, women with disability were older and had lower incomes compared to other groups. In addition to societal pressures for thinness and the misperception of the ideal body imposed on women [34], multiple overlapping and interacting social identities could account for the gendered pattern of obesity shown our results.

The prevalence of underweight and obesity was differed according to disability severity and type in this study. Mental disorder and physical disability were associated with both underweight and obesity in men and women. In addition, intellectual disability/autism, and epilepsy were associated with increased prevalence of underweight and obesity in women. Our results are consistent with previous studies, which found that persons with intellectual disability had a higher prevalence of obesity [12, 15, 35]. The association between obesity and physical disability was also reported in previous studies [13, 16, 36]. Several risk factors associated with weight gain in these population are suggested. Persons with intellectual disability may have limited control over their food selection or the amount of physical activity [12]. The quality of health care is another possible factor. Previous studies demonstrated that people with intellectual disability who lived in institutions were less likely to become obese than living with family [37, 38]. Furthermore, medications including antipsychotics, antidepressants and anticonvulsants that are frequently prescribed in persons with intellectual disability also affect weight gain [15]. Regarding physical disability, physical inactivity and muscle atrophy are risk factors for obesity [13, 39]. In contrast, previous studies have shown that specific types of disability including musculo-skeletal and respiratory disorders are risk factors for underweight in persons with disability [40].

This study has some significant implications. One of the strengths of this study is its large-scale, based on a database including about 25% of population in South Korea. To the best of our knowledge, there has been no previous study, which attempted to evaluate long-term trends in underweight and obesity among people with disability, or provided detailed analysis according to grade and types of disability.

There are some limitations to our study. First, the subjects were restricted to those who came forward for health screening, therefore people who are very old or have severe disability were more likely to be excluded due to less access to health check-ups. Second, clinical and demographic variables that may influence weight (e.g., energy intake, residence type, whether the disability is congenital or acquired) were not available from the NHID. Third, although we collected data from actual physical measurement during general health screening, in persons who cannot stand, the data might be inaccurate or unavailable.

In conclusion, the prevalence of obesity was steadily increased in people with and without disability during the recent 10 years. Obesity was especially prevalent in women with disabilities. In addition to obesity, underweight was also revealed as an important problem especially in people with severe disabilities. Both underweight and severe obesity were more prominent in people with intersecting vulnerabilities such as female and people with mental or developmental disabilities. Simultaneous efforts are needed to develop health policy to reduce both the prevalence of obesity and underweight.

## Supplementary information

Supplementary Figure

Supplementary Material
