# Supplementary figures and images for: Nationwide trends in prevalence of underweight, overweight, and obesity among people with disabilities in South Korea from 2008 to 2017
Source: Int J Obes (Lond). 2021 Dec 3;46(3):613–22. doi: 10.1038/s41366-021-01030-x (PMC8872979; doi:10.1038/s41366-021-01030-x)

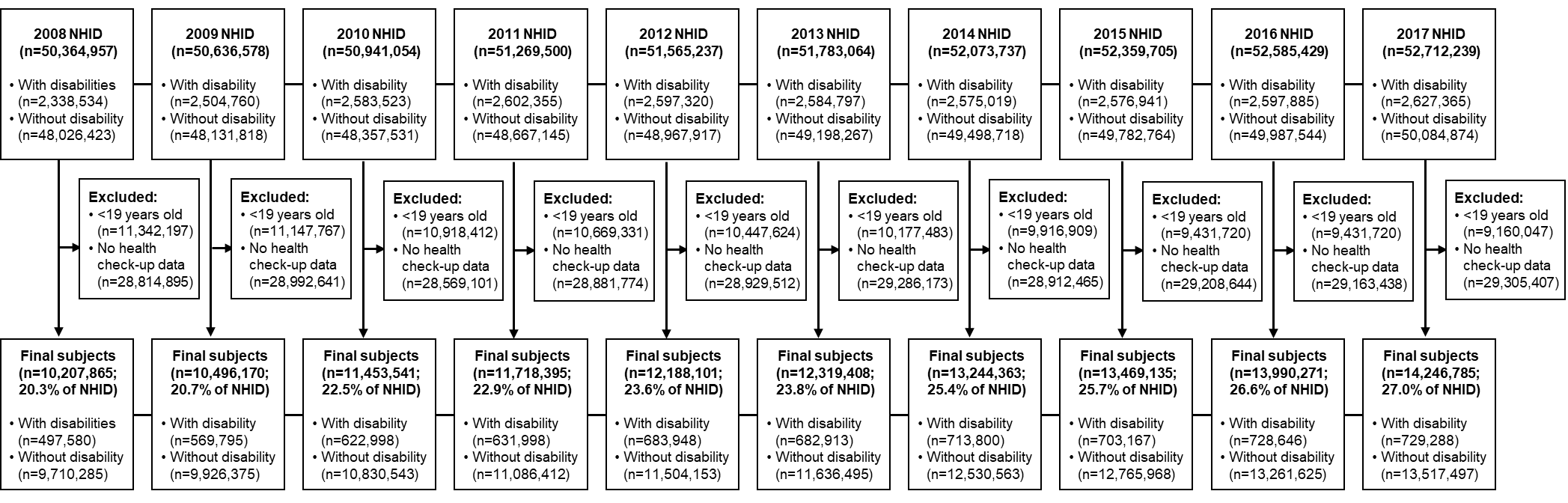

Supplement: Supplementary file 1 — Supplementary Figure [file 41366_2021_1030_MOESM1_ESM.tif]
