# Supplementary material for: Nationwide trends in prevalence of underweight, overweight, and obesity among people with disabilities in South Korea from 2008 to 2017
Source: Int J Obes (Lond). 2021 Dec 3;46(3):613–22. doi: 10.1038/s41366-021-01030-x (PMC8872979; doi:10.1038/s41366-021-01030-x)
Supplement: Supplementary file 2 — Supplementary Material [file 41366_2021_1030_MOESM2_ESM.docx]

**Figure Legends**

**Supplementary Figure 1.** Flow chart of the study population selection

**Supplementary Table 1.** Distribution of BMI and waist circumference according to disability, disability severity and type

|  | **2008** | |  | **2009** | |  | **2010** | |  | **2011** | |  | **2012** | |
| --- | --- | --- | --- | --- | --- | --- | --- | --- | --- | --- | --- | --- | --- | --- |
|  | Mean ± SD | *P* value |  | Mean ± SD | *P* value |  | Mean ± SD | *P* value |  | Mean ± SD | *P* value |  | Mean ± SD | *P* value |
| ***BMI*** |  |  |  |  |  |  |  |  |  |  |  |  |  |  |
| All subjects |  | < 0.001 |  |  | < 0.001 |  |  | < 0.001 |  |  | < 0.001 |  |  | < 0.001 |
| Without disability | 23.6 ± 3.2 |  |  | 23.7 ± 3.2 |  |  | 23.7 ± 3.2 |  |  | 23.7 ± 3.3 |  |  | 23.7 ± 3.3 |  |
| With disability | 24.2 ± 3.3 |  |  | 24.3 ± 3.3 |  |  | 24.3 ± 3.3 |  |  | 24.3 ± 3.4 |  |  | 24.3 ± 3.4 |  |
| Male |  | < 0.001 |  |  | < 0.001 |  |  | < 0.001 |  |  | < 0.001 |  |  | < 0.001 |
| Without disability | 24.1 ± 3.0 |  |  | 24.1 ± 3.1 |  |  | 24.2 ± 3.1 |  |  | 24.3 ± 3.1 |  |  | 24.3 ± 3.1 |  |
| With disability | 24.0 ± 3.1 |  |  | 24.0 ± 3.1 |  |  | 24.1 ± 3.1 |  |  | 24.1 ± 3.1 |  |  | 24.1 ± 3.2 |  |
| Female |  | < 0.001 |  |  | < 0.001 |  |  | < 0.001 |  |  | < 0.001 |  |  | < 0.001 |
| Without disability | 22.9 ± 3.3 |  |  | 23.1 ± 3.3 |  |  | 23.0 ± 3.3 |  |  | 23.1 ± 3.3 |  |  | 23.1 ± 3.4 |  |
| With disability | 24.7 ± 3.6 |  |  | 24.7 ± 3.6 |  |  | 24.7 ± 3.6 |  |  | 24.7 ± 3.7 |  |  | 24.6 ± 3.7 |  |
| Disability severity |  | < 0.001 |  |  | < 0.001 |  |  | < 0.001 |  |  | < 0.001 |  |  | < 0.001 |
| Severe (grades 1–3) | 23.8 ± 3.4 |  |  | 23.8 ± 3.5 |  |  | 23.9 ± 3.5 |  |  | 23.9 ± 3.5 |  |  | 23.9 ± 3.7 |  |
| Mild (grades 4–6) | 24.4 ± 3.2 |  |  | 24.4 ± 3.3 |  |  | 24.5 ± 3.3 |  |  | 24.5 ± 3.3 |  |  | 24.5 ± 3.3 |  |
| Disability type |  | < 0.001 |  |  | < 0.001 |  |  | < 0.001 |  |  | < 0.001 |  |  | < 0.001 |
| Physical | 24.5 ± 3.3 |  |  | 24.5 ± 3.3 |  |  | 24.5 ± 3.3 |  |  | 24.6 ± 3.3 |  |  | 24.5 ± 3.4 |  |
| Brain injury | 24.0 ± 3.2 |  |  | 24.0 ± 3.3 |  |  | 24.0 ± 3.3 |  |  | 24.0 ± 3.4 |  |  | 24.0 ± 3.4 |  |
| Facial | 23.5 ± 3.4 |  |  | 23.6 ± 3.3 |  |  | 23.6 ± 3.3 |  |  | 23.6 ± 3.3 |  |  | 23.8 ± 3.2 |  |
| Visual | 23.9 ± 3.2 |  |  | 23.9 ± 3.2 |  |  | 24.0 ± 3.2 |  |  | 24.0 ± 3.2 |  |  | 24.0 ± 3.3 |  |
| Hearing | 23.6 ± 3.1 |  |  | 23.6 ± 3.1 |  |  | 23.7 ± 3.1 |  |  | 23.7 ± 3.1 |  |  | 23.7 ± 3.1 |  |
| Language | 23.2 ± 3.2 |  |  | 23.1 ± 3.3 |  |  | 23.2 ± 3.3 |  |  | 23.1 ± 3.3 |  |  | 23.3 ± 3.3 |  |
| Intellectual | 23.6 ± 3.9 |  |  | 23.7 ± 4.0 |  |  | 23.8 ± 4.1 |  |  | 23.8 ± 4.1 |  |  | 23.7 ± 4.3 |  |
| Autism | 23.6 ± 3.9 |  |  | 24.4 ± 4.1 |  |  | 23.9 ± 4.5 |  |  | 23.9 ± 4.2 |  |  | 23.9 ± 4.2 |  |
| Mental | 25.0 ± 3.9 |  |  | 24.9 ± 3.9 |  |  | 25.0 ± 4.0 |  |  | 25.1 ± 4.0 |  |  | 24.7 ± 4.1 |  |
| Renal disease | 23.0 ± 3.1 |  |  | 23.1 ± 3.1 |  |  | 23.0 ± 3.1 |  |  | 23.0 ± 3.2 |  |  | 22.9 ± 3.2 |  |
| Heart disease | 24.1 ± 3.2 |  |  | 24.1 ± 3.2 |  |  | 24.1 ± 3.2 |  |  | 24.1 ± 3.3 |  |  | 24.0 ± 3.3 |  |
| Respiratory disease | 21.9 ± 3.5 |  |  | 22.1 ± 3.5 |  |  | 22.0 ± 3.5 |  |  | 22.0 ± 3.4 |  |  | 22.1 ± 3.7 |  |
| Liver disease | 23.7 ± 3.0 |  |  | 23.6 ± 3.0 |  |  | 23.7 ± 3.0 |  |  | 23.7 ± 3.0 |  |  | 23.7 ± 3.0 |  |
| Ostomy | 23.6 ± 3.2 |  |  | 23.7 ± 3.3 |  |  | 24.0 ± 3.3 |  |  | 24.2 ± 3.6 |  |  | 24.1 ± 3.5 |  |
| Epilepsy | 24.0 ± 3.4 |  |  | 24.3 ± 3.6 |  |  | 24.2 ± 3.7 |  |  | 24.2 ± 3.7 |  |  | 24.1 ± 3.9 |  |
|  |  |  |  |  |  |  |  |  |  |  |  |  |  |  |
| ***Waist circumference*** |  |  |  |  |  |  |  |  |  |  |  |  |  |  |
| All subjects |  | < 0.001 |  |  | < 0.001 |  |  | < 0.001 |  |  | < 0.001 |  |  | < 0.001 |
| Without disability | 80.2 ± 9.2 |  |  | 80.1 ± 9.1 |  |  | 80.0 ± 9.2 |  |  | 80.2 ± 9.2 |  |  | 80.1 ± 9.3 |  |
| With disability | 83.8 ± 8.7 |  |  | 83.5 ± 8.7 |  |  | 83.5 ± 8.7 |  |  | 83.5 ± 8.8 |  |  | 83.4 ± 9.0 |  |
| Male |  | < 0.001 |  |  | < 0.001 |  |  | < 0.001 |  |  | < 0.001 |  |  | < 0.001 |
| Without disability | 83.7 ± 7.8 |  |  | 83.4 ± 7.8 |  |  | 83.6 ± 7.8 |  |  | 83.7 ± 7.9 |  |  | 83.7 ± 8.0 |  |
| With disability | 84.8 ± 8.1 |  |  | 84.6 ± 8.1 |  |  | 84.7 ± 8.1 |  |  | 84.7 ± 8.2 |  |  | 84.6 ± 8.4 |  |
| Female |  | < 0.001 |  |  | < 0.001 |  |  | < 0.001 |  |  | < 0.001 |  |  | < 0.001 |
| Without disability | 75.8 ± 8.9 |  |  | 76.0 ± 8.8 |  |  | 75.7 ± 8.8 |  |  | 75.9 ± 8.9 |  |  | 75.9 ± 8.9 |  |
| With disability | 81.8 ± 9.3 |  |  | 81.7 ± 9.2 |  |  | 81.6 ± 9.3 |  |  | 81.5 ± 9.4 |  |  | 81.4 ± 9.5 |  |
| Disability severity |  | < 0.001 |  |  | < 0.001 |  |  | < 0.001 |  |  | < 0.001 |  |  | < 0.001 |
| Severe (grades 1–3) | 83.4 ± 9.1 |  |  | 83.0 ± 9.2 |  |  | 83.1 ± 9.2 |  |  | 83.0 ± 9.3 |  |  | 82.8 ± 9.7 |  |
| Mild (grades 4–6) | 83.9 ± 8.5 |  |  | 83.7 ± 8.5 |  |  | 83.7 ± 8.5 |  |  | 83.6 ± 8.6 |  |  | 83.6 ± 8.7 |  |
| Disability type |  | < 0.001 |  |  | < 0.001 |  |  | < 0.001 |  |  | < 0.001 |  |  | < 0.001 |
| Physical | 84.1 ± 8.6 |  |  | 83.9 ± 8.6 |  |  | 83.9 ± 8.6 |  |  | 83.9 ± 8.7 |  |  | 83.9 ± 8.8 |  |
| Brain injury | 84.6 ± 8.6 |  |  | 84.1 ± 8.7 |  |  | 84.0 ± 9.0 |  |  | 83.9 ± 9.1 |  |  | 83.9 ± 9.2 |  |
| Facial | 81.1 ± 9.6 |  |  | 80.9 ± 9.1 |  |  | 80.7 ± 9.0 |  |  | 80.7 ± 9.0 |  |  | 81.2 ± 9.0 |  |
| Visual | 83.1 ± 8.5 |  |  | 82.7 ± 8.6 |  |  | 82.7 ± 8.6 |  |  | 82.7 ± 8.7 |  |  | 82.7 ± 8.8 |  |
| Hearing | 82.8 ± 8.6 |  |  | 82.4 ± 8.6 |  |  | 82.5 ± 8.5 |  |  | 82.3 ± 8.6 |  |  | 82.3 ± 8.7 |  |
| Language | 81.4 ± 9.0 |  |  | 81.2 ± 8.9 |  |  | 81.4 ± 9.0 |  |  | 81.0 ± 9.1 |  |  | 81.2 ± 9.1 |  |
| Intellectual | 80.9 ± 10.0 |  |  | 80.9 ± 10.2 |  |  | 80.9 ± 10.2 |  |  | 80.8 ± 10.3 |  |  | 80.4 ± 10.9 |  |
| Autism | 81.7 ± 10.8 |  |  | 84.0 ± 11.8 |  |  | 82.4 ± 11.3 |  |  | 81.7 ± 10.5 |  |  | 82.1 ± 11.0 |  |
| Mental | 84.8 ± 9.9 |  |  | 84.3 ± 9.9 |  |  | 84.4 ± 10.2 |  |  | 84.7 ± 10.3 |  |  | 84.0 ± 10.5 |  |
| Renal disease | 81.4 ± 9.1 |  |  | 81.2 ± 9.3 |  |  | 80.9 ± 9.3 |  |  | 81.0 ± 9.4 |  |  | 80.8 ± 9.5 |  |
| Heart disease | 84.6 ± 9.1 |  |  | 84.2 ± 9.2 |  |  | 84.0 ± 9.0 |  |  | 84.2 ± 9.5 |  |  | 83.9 ± 9.5 |  |
| Respiratory disease | 81.8 ± 9.9 |  |  | 81.9 ± 10.0 |  |  | 81.9 ± 10.0 |  |  | 81.5 ± 10.0 |  |  | 81.8 ± 10.2 |  |
| Liver disease | 84.1 ± 8.1 |  |  | 83.5 ± 8.6 |  |  | 83.8 ± 8.3 |  |  | 83.4 ± 8.2 |  |  | 83.6 ± 8.3 |  |
| Ostomy | 83.3 ± 8.7 |  |  | 83.0 ± 8.5 |  |  | 83.3 ± 8.5 |  |  | 83.4 ± 8.6 |  |  | 83.4 ± 8.8 |  |
| Epilepsy | 81.5 ± 9.2 |  |  | 81.4 ± 9.7 |  |  | 81.4 ± 9.7 |  |  | 81.4 ± 9.8 |  |  | 81.4 ± 10.1 |  |

Abbreviation: SD, standard deviation; BMI, Body mass index.

*P* values were calculated using Student’s *t*-test or one-way analysis of variance.

**Supplementary Table 1.** Continued

|  | **2013** | |  | **2014** | |  | **2015** | |  | **2016** | |  | **2017** | |
| --- | --- | --- | --- | --- | --- | --- | --- | --- | --- | --- | --- | --- | --- | --- |
|  | Mean ± SD | *P* value |  | Mean ± SD | *P* value |  | Mean ± SD | *P* value |  | Mean ± SD | *P* value |  | Mean ± SD | *P* value |
| ***BMI*** |  |  |  |  |  |  |  |  |  |  |  |  |  |  |
| All subjects |  | < 0.001 |  |  | < 0.001 |  |  | < 0.001 |  |  | < 0.001 |  |  | < 0.001 |
| Without disability | 23.8 ± 3.3 |  |  | 23.8 ± 3.4 |  |  | 23.9 ± 3.4 |  |  | 24.0 ± 3.5 |  |  | 24.1 ± 3.5 |  |
| With disability | 24.3 ± 3.5 |  |  | 24.3 ± 3.5 |  |  | 24.4 ± 3.5 |  |  | 24.5 ± 3.5 |  |  | 24.6 ± 3.6 |  |
| Male |  | < 0.001 |  |  | < 0.001 |  |  | < 0.001 |  |  | < 0.001 |  |  | < 0.001 |
| Without disability | 24.3 ± 3.2 |  |  | 24.4 ± 3.2 |  |  | 24.6 ± 3.2 |  |  | 24.7 ± 3.3 |  |  | 24.8 ± 3.3 |  |
| With disability | 24.1 ± 3.2 |  |  | 24.1 ± 3.3 |  |  | 24.3 ± 3.3 |  |  | 24.3 ± 3.3 |  |  | 24.5 ± 3.3 |  |
| Female |  | < 0.001 |  |  | < 0.001 |  |  | < 0.001 |  |  | < 0.001 |  |  | < 0.001 |
| Without disability | 23.1 ± 3.4 |  |  | 23.1 ± 3.4 |  |  | 23.2 ± 3.5 |  |  | 23.3 ± 3.5 |  |  | 23.4 ± 3.5 |  |
| With disability | 24.6 ± 3.8 |  |  | 24.6 ± 3.8 |  |  | 24.7 ± 3.8 |  |  | 24.7 ± 3.8 |  |  | 24.8 ± 3.9 |  |
| Disability severity |  | < 0.001 |  |  | < 0.001 |  |  | < 0.001 |  |  | < 0.001 |  |  | < 0.001 |
| Severe (grades 1–3) | 23.9 ± 3.7 |  |  | 23.9 ± 3.8 |  |  | 24.0 ± 3.8 |  |  | 24.1 ± 3.9 |  |  | 24.3 ± 3.9 |  |
| Mild (grades 4–6) | 24.4 ± 3.3 |  |  | 24.5 ± 3.3 |  |  | 24.6 ± 3.4 |  |  | 24.6 ± 3.4 |  |  | 24.7 ± 3.4 |  |
| Disability type |  | < 0.001 |  |  | < 0.001 |  |  | < 0.001 |  |  | < 0.001 |  |  | < 0.001 |
| Physical | 24.5 ± 3.4 |  |  | 24.6 ± 3.4 |  |  | 24.7 ± 3.4 |  |  | 24.7 ± 3.5 |  |  | 24.8 ± 3.5 |  |
| Brain injury | 24.0 ± 3.4 |  |  | 24.0 ± 3.5 |  |  | 24.1 ± 3.5 |  |  | 24.2 ± 3.5 |  |  | 24.3 ± 3.6 |  |
| Facial | 23.8 ± 3.4 |  |  | 23.8 ± 3.4 |  |  | 24.0 ± 3.5 |  |  | 23.9 ± 3.4 |  |  | 24.2 ± 3.5 |  |
| Visual | 24.0 ± 3.3 |  |  | 24.0 ± 3.3 |  |  | 24.2 ± 3.3 |  |  | 24.2 ± 3.4 |  |  | 24.3 ± 3.4 |  |
| Hearing | 23.7 ± 3.2 |  |  | 23.8 ± 3.2 |  |  | 23.8 ± 3.2 |  |  | 23.9 ± 3.2 |  |  | 24.0 ± 3.3 |  |
| Language | 23.4 ± 3.5 |  |  | 23.4 ± 3.3 |  |  | 23.6 ± 3.5 |  |  | 23.6 ± 3.5 |  |  | 23.7 ± 3.6 |  |
| Intellectual | 23.8 ± 4.4 |  |  | 23.9 ± 4.4 |  |  | 24.0 ± 4.5 |  |  | 24.1 ± 4.6 |  |  | 24.4 ± 4.6 |  |
| Autism | 23.9 ± 4.3 |  |  | 24.1 ± 4.4 |  |  | 24.5 ± 4.7 |  |  | 24.7 ± 4.7 |  |  | 24.8 ± 4.7 |  |
| Mental | 24.8 ± 4.2 |  |  | 24.8 ± 4.2 |  |  | 25.0 ± 4.3 |  |  | 25.1 ± 4.3 |  |  | 25.4 ± 4.4 |  |
| Renal disease | 23.0 ± 3.2 |  |  | 23.0 ± 3.3 |  |  | 23.1 ± 3.3 |  |  | 23.2 ± 3.3 |  |  | 23.3 ± 3.4 |  |
| Heart disease | 24.0 ± 3.4 |  |  | 23.8 ± 3.4 |  |  | 24.0 ± 3.5 |  |  | 23.9 ± 3.5 |  |  | 24.0 ± 3.6 |  |
| Respiratory disease | 21.9 ± 3.5 |  |  | 22.1 ± 3.6 |  |  | 22.0 ± 3.5 |  |  | 22.1 ± 3.7 |  |  | 22.2 ± 3.6 |  |
| Liver disease | 23.7 ± 3.2 |  |  | 23.6 ± 3.0 |  |  | 23.7 ± 3.1 |  |  | 23.7 ± 3.1 |  |  | 23.8 ± 3.2 |  |
| Ostomy | 24.1 ± 3.5 |  |  | 24.1 ± 3.5 |  |  | 24.0 ± 3.4 |  |  | 24.0 ± 3.5 |  |  | 24.2 ± 3.5 |  |
| Epilepsy | 24.2 ± 3.9 |  |  | 24.2 ± 3.9 |  |  | 24.4 ± 3.9 |  |  | 24.4 ± 4.0 |  |  | 24.8 ± 4.1 |  |
|  |  |  |  |  |  |  |  |  |  |  |  |  |  |  |
| ***Waist circumference*** |  |  |  |  |  |  |  |  |  |  |  |  |  |  |
| All subjects |  | < 0.001 |  |  | < 0.001 |  |  | < 0.001 |  |  | < 0.001 |  |  | < 0.001 |
| Without disability | 80.3 ± 9.4 |  |  | 80.4 ± 9.4 |  |  | 80.7 ± 9.6 |  |  | 80.9 ± 9.7 |  |  | 81.1 ± 9.8 |  |
| With disability | 83.4 ± 9.1 |  |  | 83.5 ± 9.2 |  |  | 83.9 ± 9.3 |  |  | 84.1 ± 9.3 |  |  | 84.3 ± 9.4 |  |
| Male |  | < 0.001 |  |  | < 0.001 |  |  | < 0.001 |  |  | < 0.001 |  |  | < 0.001 |
| Without disability | 83.9 ± 8.1 |  |  | 84.1 ± 8.1 |  |  | 84.5 ± 8.2 |  |  | 84.8 ± 8.3 |  |  | 85.1 ± 8.4 |  |
| With disability | 84.7 ± 8.5 |  |  | 84.8 ± 8.6 |  |  | 85.2 ± 8.7 |  |  | 85.4 ± 8.7 |  |  | 85.7 ± 8.8 |  |
| Female |  | < 0.001 |  |  | < 0.001 |  |  | < 0.001 |  |  | < 0.001 |  |  | < 0.001 |
| Without disability | 76.1 ± 9.0 |  |  | 76.1 ± 9.0 |  |  | 76.4 ± 9.1 |  |  | 76.5 ± 9.2 |  |  | 76.7 ± 9.3 |  |
| With disability | 81.4 ± 9.6 |  |  | 81.5 ± 9.7 |  |  | 81.7 ± 9.8 |  |  | 81.9 ± 9.9 |  |  | 82.0 ± 9.9 |  |
| Disability severity |  | < 0.001 |  |  | < 0.001 |  |  | < 0.001 |  |  | < 0.001 |  |  | < 0.001 |
| Severe (grades 1–3) | 82.9 ± 9.8 |  |  | 83.0 ± 9.9 |  |  | 83.2 ± 10.1 |  |  | 83.5 ± 10.2 |  |  | 83.8 ± 10.3 |  |
| Mild (grades 4–6) | 83.7 ± 8.8 |  |  | 83.8 ± 8.8 |  |  | 84.1 ± 8.9 |  |  | 84.3 ± 9.0 |  |  | 84.5 ± 9.1 |  |
| Disability type |  | < 0.001 |  |  | < 0.001 |  |  | < 0.001 |  |  | < 0.001 |  |  | < 0.001 |
| Physical | 83.9 ± 8.9 |  |  | 84.0 ± 8.9 |  |  | 84.4 ± 9.0 |  |  | 84.6 ± 9.1 |  |  | 84.8 ± 9.1 |  |
| Brain injury | 84.0 ± 9.3 |  |  | 84.0 ± 9.4 |  |  | 84.3 ± 9.5 |  |  | 84.6 ± 9.5 |  |  | 84.8 ± 9.6 |  |
| Facial | 81.2 ± 9.3 |  |  | 81.2 ± 9.2 |  |  | 81.5 ± 9.5 |  |  | 81.5 ± 9.2 |  |  | 82.5 ± 9.6 |  |
| Visual | 82.7 ± 8.9 |  |  | 82.8 ± 9.0 |  |  | 83.1 ± 9.0 |  |  | 83.4 ± 9.1 |  |  | 83.6 ± 9.2 |  |
| Hearing | 82.3 ± 8.8 |  |  | 82.5 ± 8.9 |  |  | 82.7 ± 9.0 |  |  | 83.0 ± 9.0 |  |  | 83.2 ± 9.1 |  |
| Language | 81.4 ± 9.4 |  |  | 81.6 ± 9.3 |  |  | 82.0 ± 9.4 |  |  | 82.1 ± 9.7 |  |  | 82.4 ± 9.7 |  |
| Intellectual | 80.9 ± 11.0 |  |  | 81.2 ± 11.2 |  |  | 81.4 ± 11.4 |  |  | 81.8 ± 11.5 |  |  | 82.3 ± 11.6 |  |
| Autism | 82.6 ± 11.2 |  |  | 82.6 ± 11.3 |  |  | 84.1 ± 12.1 |  |  | 84.3 ± 11.5 |  |  | 84.9 ± 11.9 |  |
| Mental | 84.5 ± 10.7 |  |  | 84.7 ± 10.8 |  |  | 85.2 ± 11.0 |  |  | 85.3 ± 11.1 |  |  | 86.1 ± 11.1 |  |
| Renal disease | 80.9 ± 9.6 |  |  | 81.0 ± 9.7 |  |  | 81.2 ± 9.8 |  |  | 81.5 ± 9.8 |  |  | 81.8 ± 10.0 |  |
| Heart disease | 83.8 ± 9.9 |  |  | 83.5 ± 9.6 |  |  | 83.8 ± 10.1 |  |  | 83.8 ± 9.9 |  |  | 84.1 ± 10.4 |  |
| Respiratory disease | 81.4 ± 10.1 |  |  | 81.6 ± 10.3 |  |  | 81.4 ± 10.2 |  |  | 81.5 ± 10.5 |  |  | 81.6 ± 10.5 |  |
| Liver disease | 83.5 ± 8.8 |  |  | 83.4 ± 8.7 |  |  | 83.8 ± 8.8 |  |  | 83.5 ± 8.6 |  |  | 84.0 ± 8.9 |  |
| Ostomy | 83.6 ± 8.9 |  |  | 83.4 ± 9.0 |  |  | 83.5 ± 9.0 |  |  | 83.5 ± 9.2 |  |  | 83.7 ± 9.2 |  |
| Epilepsy | 81.6 ± 10.2 |  |  | 81.9 ± 10.2 |  |  | 82.1 ± 10.6 |  |  | 82.3 ± 10.5 |  |  | 83.3 ± 10.9 |  |

Abbreviation: SD, standard deviation; BMI, Body mass index

*P* values were calculated using Student’s *t*-test or one-way analysis of variance.

**Supplementary Table 2.** Age-standardized rates of underweight and obesity from 2008 to 2017 by disability

|  |  | **2008** | **2009** | **2010** | | **2011** | **2012** | **2013** | **2014** | **2015** | **2016** | **2017** |
| --- | --- | --- | --- | --- | --- | --- | --- | --- | --- | --- | --- | --- |
| ***Underweight (BMI <18.5 kg/m^2^), %*** | | | | | | | | | | | | |
| All - | without disability | 5.3 | 5.3 | 5.5 | | 5.3 | 5.4 | 5.3 | 5.3 | 5.0 | 4.9 | 4.5 |
|  | with disability | 5.7 | 5.6 | 5.9 | | 5.7 | 6.6 | 6.6 | 6.6 | 6.7 | 6.3 | 6.2 |
| Men - | without disability | 2.5 | 2.5 | 2.5 | | 2.4 | 2.4 | 2.3 | 2.3 | 2.1 | 2.1 | 1.9 |
|  | with disability | 4.0 | 4.0 | 4.2 | | 3.9 | 4.8 | 5.1 | 5.0 | 5.0 | 4.8 | 4.8 |
| Women - | without disability | 8.1 | 8.0 | 8.4 | | 8.2 | 8.3 | 8.2 | 8.2 | 7.8 | 7.7 | 7.0 |
|  | with disability | 7.3 | 7.2 | 7.6 | | 7.4 | 8.4 | 8.1 | 8.2 | 8.2 | 7.9 | 7.7 |
| ***Obesity (BMI ≥25.0 kg/m^2^), %*** | | | | | | | | | | | | |
| All - | without disability | 28.4 | 28.8 | 28.8 | | 29.5 | 29.5 | 30.1 | 30.5 | 32.0 | 32.9 | 34.3 |
|  | with disability | 33.5 | 34.8 | 34.7 | | 35.3 | 35.3 | 36.2 | 36.8 | 37.9 | 39.3 | 40.6 |
| Men - | without disability | 35.2 | 35.3 | 36.0 | | 36.8 | 37.1 | 38.0 | 38.8 | 40.8 | 42.2 | 44.0 |
|  | with disability | 36.6 | 37.6 | 37.8 | | 38.6 | 38.2 | 38.9 | 39.9 | 41.5 | 42.9 | 44.4 |
| Women - | without disability | 21.8 | 22.4 | 21.8 | | 22.4 | 22.0 | 22.5 | 22.5 | 23.3 | 23.9 | 24.8 |
|  | with disability | 30.5 | 32.0 | 31.6 | | 32.1 | 32.5 | 33.5 | 33.8 | 34.5 | 35.7 | 36.8 |
| ***Severe obesity (BMI ≥30.0 kg/m^2^), %*** | | | | |  | | | | | | | |
| All - | without disability | 3.1 | 3.3 | 3.4 | | 3.7 | 3.8 | 4.2 | 4.4 | 5.0 | 5.4 | 5.9 |
|  | with disability | 4.9 | 5.7 | 5.7 | | 6.2 | 6.6 | 7.1 | 7.5 | 8.1 | 8.8 | 9.6 |
| Men - | without disability | 3.5 | 3.7 | 4.0 | | 4.3 | 4.5 | 4.9 | 5.3 | 6.0 | 6.5 | 7.2 |
|  | with disability | 4.5 | 5.0 | 5.3 | | 5.8 | 5.9 | 6.4 | 6.8 | 7.6 | 8.4 | 9.2 |
| Women - | without disability | 2.7 | 2.9 | 2.9 | | 3.2 | 3.2 | 3.5 | 3.6 | 3.9 | 4.3 | 4.6 |
|  | with disability | 5.3 | 6.3 | 6.2 | | 6.7 | 7.3 | 7.8 | 8.2 | 8.5 | 9.2 | 9.9 |
| ***Abdominal obesity (M: WC ≥90 cm, F: WC ≥85 cm), %*** | | |  |  | |  |  |  |  |  |  |  |
| All - | without disability | 17.5 | 16.7 | 16.6 | | 17.1 | 17.1 | 17.9 | 18.2 | 19.6 | 20.3 | 21.1 |
|  | with disability | 22.3 | 22.2 | 22.0 | | 22.6 | 23.1 | 24.2 | 25.1 | 26.1 | 27.4 | 28.5 |
| Men - | without disability | 20.2 | 19.3 | 19.5 | | 20.1 | 20.2 | 21.3 | 21.8 | 23.7 | 24.7 | 26.0 |
|  | with disability | 23.2 | 22.8 | 22.7 | | 23.5 | 23.7 | 24.5 | 25.6 | 27.2 | 28.7 | 30.0 |
| Women - | without disability | 14.8 | 14.3 | 13.8 | | 14.2 | 14.1 | 14.7 | 14.7 | 15.6 | 16.0 | 16.3 |
|  | with disability | 21.4 | 21.6 | 21.4 | | 21.7 | 22.5 | 23.8 | 24.6 | 25.0 | 26.1 | 27.0 |

Abbreviation: BMI, Body mass index; WC, Waist circumference.

**Supplementary Table 3**. Age-standardized rates (ASR) of underweight and obesity by disability in the most recent year with available data (2017)

|  | **Underweight**  (BMI <18.5 kg/m^2^) | | |  | **Obesity**  (BMI ≥25.0 kg/m^2^) | | |  | **Severe obesity**  (BMI ≥30.0 kg/m^2^) | | |  | **Abdominal obesity**  (waist circumstance ≥90 cm or ≥85 cm) | | |
| --- | --- | --- | --- | --- | --- | --- | --- | --- | --- | --- | --- | --- | --- | --- | --- |
|  | Total | Male | Female |  | Total | Male | Female |  | Total | Male | Female |  | Total | Male | Female |
| All subjects |  |  |  |  |  |  |  |  |  |  |  |  |  |  |  |
| Without disability | 4.5 | 1.9 | 7.0 |  | 34.3 | 44.0 | 24.8 |  | 5.9 | 7.2 | 4.6 |  | 21.1 | 26.0 | 16.3 |
| With disability | 6.2 | 4.8 | 7.7 |  | 40.6 | 44.4 | 36.8 |  | 9.6 | 9.2 | 9.9 |  | 28.5 | 30.0 | 27.0 |
| Disability severity |  |  |  |  |  |  |  |  |  |  |  |  |  |  |  |
| Severe (grades 1–3) | 7.7 | 6.9 | 8.5 |  | 39.7 | 40.3 | 39.2 |  | 10.2 | 8.8 | 11.6 |  | 29.9 | 29.5 | 30.4 |
| Mild (grades 4–6) | 5.1 | 2.6 | 7.5 |  | 39.3 | 46.9 | 31.8 |  | 8.4 | 9.5 | 7.2 |  | 25.9 | 30.0 | 22.0 |
| Disability type |  |  |  |  |  |  |  |  |  |  |  |  |  |  |  |
| Physical | 6.2 | 4.2 | 8.1 |  | 39.8 | 46.5 | 33.2 |  | 8.9 | 9.7 | 8.2 |  | 26.8 | 30.2 | 23.6 |
| Brain injury | 9.8 | 6.8 | 12.7 |  | 35.9 | 40.4 | 31.4 |  | 7.5 | 7.5 | 7.5 |  | 27.7 | 29.6 | 25.8 |
| Facial | 4.7 | 1.7 | 7.6 |  | 30.5 | 37.2 | 24.0 |  | 5.9 | 6.4 | 5.5 |  | 21.2 | 23.4 | 19.1 |
| Visual | 5.1 | 2.7 | 7.4 |  | 37.8 | 44.5 | 31.2 |  | 7.9 | 8.6 | 7.2 |  | 24.5 | 28.4 | 20.8 |
| Hearing | 5.7 | 3.8 | 7.6 |  | 34.1 | 40.0 | 28.3 |  | 5.9 | 6.5 | 5.4 |  | 22.0 | 25.0 | 19.1 |
| Language | 8.0 | 5.3 | 10.7 |  | 36.3 | 35.7 | 37.0 |  | 8.7 | 6.9 | 10.4 |  | 23.7 | 23.6 | 23.9 |
| Intellectual | 8.5 | 8.6 | 8.5 |  | 41.1 | 39.1 | 43.1 |  | 11.5 | 9.3 | 13.8 |  | 31.3 | 28.2 | 34.3 |
| Autism | 8.1 | 10.4 | 5.9 |  | 35.9 | 41.4 | 30.6 |  | 11.7 | 11.2 | 12.1 |  | 31.8 | 32.4 | 31.3 |
| Mental | 5.2 | 5.8 | 4.7 |  | 52.9 | 50.4 | 55.3 |  | 18.0 | 16.5 | 19.4 |  | 45.7 | 43.7 | 47.5 |
| Renal disease | 10.0 | 4.6 | 15.3 |  | 26.6 | 32.2 | 21.2 |  | 6.5 | 7.4 | 5.6 |  | 21 | 24.7 | 17.4 |
| Heart disease | 12.5 | 7.3 | 17.6 |  | 28.6 | 35.3 | 22.0 |  | 7.0 | 9.5 | 4.6 |  | 23.8 | 32.1 | 15.7 |
| Respiratory disease | 20.2 | 12.1 | 28.1 |  | 13.9 | 14.8 | 13.1 |  | 1.6 | 1.7 | 1.4 |  | 13.2 | 13.1 | 13.2 |
| Liver disease | 9.8 | 9.8 | 9.8 |  | 25.4 | 29.5 | 21.5 |  | 5.0 | 4.9 | 5.0 |  | 20.8 | 22.7 | 18.9 |
| Ostomy | 8.7 | 3.1 | 14.1 |  | 38.9 | 44.3 | 33.6 |  | 11.2 | 16.5 | 6.1 |  | 24.7 | 27.1 | 22.4 |
| Epilepsy | 6.2 | 1.2 | 11.0 |  | 42.3 | 45.6 | 39.0 |  | 11.5 | 12.3 | 10.7 |  | 32.1 | 35.4 | 28.9 |

Abbreviation: BMI, Body mass index

**Supplementary Table 4.** Crude rates of underweight and obesity by disability in the most recent year with available data (2017)

|  | **Total** | |  | **Male** | |  | **Female** | |
| --- | --- | --- | --- | --- | --- | --- | --- | --- |
|  | n | Crude rate (%) |  | n | Crude rate (%) |  | n | Crude rate (%) |
| ***Underweight (BMI <18.5 kg/m^2^)*** | | | | | | | | |
| All subjects |  |  |  |  |  |  |  |  |
| Without disability | 425,278 | 3.2 |  | 111,882 | 1.6 |  | 313,396 | 4.9 |
| With disability | 22,864 | 3.1 |  | 13,241 | 2.9 |  | 9,623 | 3.5 |
| Disability severity |  |  |  |  |  |  |  |  |
| Severe (grades 1–3) | 10,543 | 5.3 |  | 6,149 | 4.9 |  | 4,394 | 5.9 |
| Mild (grades 4–6) | 12,321 | 2.3 |  | 7,092 | 2.2 |  | 5,229 | 2.6 |
| Disability type |  |  |  |  |  |  |  |  |
| Physical | 10,216 | 2.3 |  | 6,204 | 2.3 |  | 4,012 | 2.4 |
| Brain injury | 1,782 | 4.3 |  | 834 | 3.2 |  | 948 | 6.2 |
| Facial | 31 | 3.2 |  | 13 | 2.2 |  | 18 | 4.8 |
| Visual | 2,295 | 2.9 |  | 1,235 | 2.5 |  | 1,060 | 3.7 |
| Hearing | 3,147 | 3.7 |  | 1,741 | 3.5 |  | 1,406 | 4.2 |
| Language | 235 | 5.0 |  | 150 | 4.3 |  | 85 | 6.9 |
| Intellectual | 2,727 | 8.2 |  | 1,740 | 8.5 |  | 987 | 7.7 |
| Autism | 58 | 6.0 |  | 49 | 5.7 |  | 9 | 8.9 |
| Mental | 739 | 4.1 |  | 420 | 4.7 |  | 319 | 3.5 |
| Renal disease | 798 | 5.3 |  | 329 | 3.5 |  | 469 | 8.4 |
| Heart disease | 83 | 5.0 |  | 40 | 3.6 |  | 43 | 8.0 |
| Respiratory disease | 423 | 15.1 |  | 318 | 15.1 |  | 105 | 15.0 |
| Liver disease | 107 | 3.5 |  | 67 | 2.9 |  | 40 | 5.2 |
| Ostomy | 153 | 4.3 |  | 85 | 3.8 |  | 68 | 5.2 |
| Epilepsy | 70 | 3.9 |  | 16 | 1.7 |  | 54 | 6.4 |
| ***Obesity (BMI ≥25.0 kg/m^2^)*** | | | | | | | | |
| All subjects |  |  |  |  |  |  |  |  |
| Without disability | 5,021,478 | 37.2 |  | 3,200,422 | 45.0 |  | 1,821,056 | 28.5 |
| With disability | 315,096 | 43.2 |  | 189,334 | 42.0 |  | 125,762 | 45.1 |
| Disability severity |  |  |  |  |  |  |  |  |
| Severe (grades 1–3) | 79,839 | 39.9 |  | 48,729 | 38.7 |  | 31,110 | 41.8 |
| Mild (grades 4–6) | 235,257 | 44.5 |  | 140,605 | 43.3 |  | 94,652 | 46.3 |
| Disability type |  |  |  |  |  |  |  |  |
| Physical | 201,936 | 45.9 |  | 120,009 | 44.1 |  | 81,927 | 48.9 |
| Brain injury | 16,955 | 41.1 |  | 10,868 | 41.8 |  | 6,087 | 39.9 |
| Facial | 359 | 37.3 |  | 229 | 38.8 |  | 130 | 34.9 |
| Visual | 31,431 | 40.3 |  | 20,355 | 41.3 |  | 11,076 | 38.5 |
| Hearing | 30,717 | 36.5 |  | 18,393 | 36.5 |  | 12,324 | 36.4 |
| Language | 1,583 | 33.7 |  | 1,198 | 34.6 |  | 385 | 31.2 |
| Intellectual | 13,844 | 41.7 |  | 8,072 | 39.6 |  | 5,772 | 44.9 |
| Autism | 433 | 44.9 |  | 394 | 45.6 |  | 39 | 38.6 |
| Mental | 9,266 | 51.4 |  | 4,150 | 46.5 |  | 5,116 | 56.2 |
| Renal disease | 4,201 | 28.1 |  | 2,754 | 29.4 |  | 1,447 | 25.9 |
| Heart disease | 613 | 37.2 |  | 451 | 40.7 |  | 162 | 30.1 |
| Respiratory disease | 574 | 20.4 |  | 407 | 19.3 |  | 167 | 23.8 |
| Liver disease | 1,017 | 33.1 |  | 773 | 33.6 |  | 244 | 31.8 |
| Ostomy | 1,377 | 38.9 |  | 841 | 37.7 |  | 536 | 40.8 |
| Epilepsy | 790 | 43.6 |  | 440 | 45.4 |  | 350 | 41.4 |
| ***Severe obesity (BMI ≥30.0 kg/m^2^)*** | | | | | | | | |
| All subjects |  |  |  |  |  |  |  |  |
| Without disability | 747,241 | 5.5 |  | 451,439 | 6.3 |  | 295,802 | 4.6 |
| With disability | 49,307 | 6.8 |  | 23,631 | 5.3 |  | 25,676 | 9.2 |
| Disability severity |  |  |  |  |  |  |  |  |
| Severe (grades 1–3) | 14,756 | 7.4 |  | 7,269 | 5.8 |  | 7,487 | 10.1 |
| Mild (grades 4–6) | 34,551 | 6.5 |  | 16,362 | 5.0 |  | 18,189 | 8.9 |
| Disability type |  |  |  |  |  |  |  |  |
| Physical | 31,628 | 7.2 |  | 14,658 | 5.4 |  | 16,970 | 10.1 |
| Brain injury | 2,306 | 5.6 |  | 1,241 | 4.8 |  | 1,065 | 7.0 |
| Facial | 55 | 5.7 |  | 33 | 5.6 |  | 22 | 5.9 |
| Visual | 4,207 | 5.4 |  | 2,367 | 4.8 |  | 1,840 | 6.4 |
| Hearing | 3,244 | 3.9 |  | 1,600 | 3.2 |  | 1,644 | 4.9 |
| Language | 217 | 4.6 |  | 144 | 4.2 |  | 73 | 5.9 |
| Intellectual | 3,764 | 11.3 |  | 1,914 | 9.4 |  | 1,850 | 14.4 |
| Autism | 126 | 13.1 |  | 112 | 13.0 |  | 14 | 13.9 |
| Mental | 2,573 | 14.3 |  | 886 | 9.9 |  | 1,687 | 18.5 |
| Renal disease | 571 | 3.8 |  | 334 | 3.6 |  | 237 | 4.2 |
| Heart disease | 91 | 5.5 |  | 65 | 5.9 |  | 26 | 4.8 |
| Respiratory disease | 64 | 2.3 |  | 36 | 1.7 |  | 28 | 4.0 |
| Liver disease | 116 | 3.8 |  | 76 | 3.3 |  | 40 | 5.2 |
| Ostomy | 171 | 4.8 |  | 83 | 3.7 |  | 88 | 6.7 |
| Epilepsy | 174 | 9.6 |  | 82 | 8.5 |  | 92 | 10.9 |
| ***Abdominal obesity (M: WC ≥90 cm, F: WC ≥85 cm)*** | | | | | | | | |
| All subjects |  |  |  |  |  |  |  |  |
| Without disability | 3,171,040 | 23.5 |  | 1,951,289 | 27.4 |  | 1,219,751 | 19.1 |
| With disability | 248,225 | 34.0 |  | 141,083 | 31.3 |  | 107,142 | 38.4 |
| Disability severity |  |  |  |  |  |  |  |  |
| Severe (grades 1–3) | 66,101 | 33.0 |  | 39,211 | 31.2 |  | 26,890 | 36.1 |
| Mild (grades 4–6) | 182,124 | 34.4 |  | 101,872 | 31.4 |  | 80,252 | 39.2 |
| Disability type |  |  |  |  |  |  |  |  |
| Physical | 156,461 | 35.6 |  | 87,109 | 32.0 |  | 69,352 | 41.4 |
| Brain injury | 15,012 | 36.4 |  | 9,152 | 35.2 |  | 5,860 | 38.4 |
| Facial | 258 | 26.8 |  | 162 | 27.5 |  | 96 | 25.7 |
| Visual | 23,750 | 30.5 |  | 14,641 | 29.7 |  | 9,109 | 31.7 |
| Hearing | 25,271 | 30.0 |  | 14,521 | 28.8 |  | 10,750 | 31.8 |
| Language | 1,189 | 25.3 |  | 892 | 25.8 |  | 297 | 24.1 |
| Intellectual | 10,277 | 30.9 |  | 5,726 | 28.1 |  | 4,551 | 35.4 |
| Autism | 3320 | 34.4 |  | 300 | 34.7 |  | 32 | 31.7 |
| Mental | 8,104 | 45.0 |  | 3,594 | 40.3 |  | 4,510 | 49.5 |
| Renal disease | 3,734 | 25.0 |  | 2,377 | 25.4 |  | 1,357 | 24.3 |
| Heart disease | 552 | 33.5 |  | 412 | 37.2 |  | 140 | 26.0 |
| Respiratory disease | 700 | 24.9 |  | 524 | 24.8 |  | 176 | 25.1 |
| Liver disease | 870 | 28.3 |  | 662 | 28.8 |  | 208 | 27.1 |
| Ostomy | 1,143 | 32.3 |  | 686 | 30.8 |  | 457 | 34.8 |
| Epilepsy | 572 | 31.5 |  | 325 | 33.5 |  | 247 | 29.2 |

Abbreviation: BMI, Body mass index; WC, Waist circumference.
